# Unpacking the role of Chinese EFL teacher aggression and burnout in their professional success: A teachers’ psychology perspective

**DOI:** 10.3389/fpsyg.2022.1001252

**Published:** 2022-09-15

**Authors:** Dan Yang

**Affiliations:** School of Foreign Languages, Yancheng Teachers University, Yancheng, China

**Keywords:** teacher aggression, teacher burnout, professional success, Chinese EFL teachers’, regression analyses

## Abstract

This study aims to investigate the role of Chinese English as a foreign language (EFL) teachers’ aggression and burnout in their professional success. To accomplish this, 362 EFL teachers (i.e., 59 males, 303 females) were invited to respond to three valid measures of the variables (i.e., Maslach burnout inventory, teacher aggression scale, and teacher professional success scale). Performing Spearman’s rho correlation tests, negative and significant correlations were found between teacher burnout, teacher aggression, and teacher professional success. Moreover, as the results of regression analyses indicated, both teacher aggression and teacher burnout were found to be the negative predictors of EFL teachers’ professional success. It implies that the higher the amount of teacher aggression and teacher burnout, the less professionally successful a teacher would be. The implications of the results are finally discussed.

## Introduction

Without any doubt, the importance of psychological variables in teachers’ professional development could be of great importance. For example, a study conducted by Zhang et al. (2021) showed that motivation is a crucial factor for teachers that allows them to professionally develop. But what type of motivation is mentioned in the study? Teachers’ motivation to take part in professional learning is an important factor, considering their professional development. These factors were looked at from two aspects: the teacher level and the school level. At the teacher level, teachers’ prior experience with learning activities, teaching experience, self-efficacy, and conceptions of learning could be regarded as important, and at the school level, work and emotional pressure, colleague support, and principal leadership were associated with their motivation to take part in professional learning, leading to their professional development (Yang, 2021). Hence, all psychological variables have a paramount effect on teachers’ professional development.

Teachers are perceived as the main pillars of educational systems in that many factors are affected by them (Coombe, 2014). Teacher success has attracted attention in the pedagogical realm. Teacher professional success can be conceptualized as the extent to which a teacher obtains a sense of achievement (Hung et al., 2007). According to this definition, this sense of achievement comprises boosting skills, increasing knowledge, and modifying behavior. In addition, teachers’ success is influenced by teachers’ attitudes, worries, and expectations (Hung et al., 2007). According to Derakhshan et al. (2020b) teachers’ success is impacted by two factors, teachers’ positive attitudes and their constant professional development. Likewise, teachers’ autonomy and their professional identity are great predictors of teacher success (Derakhshan et al., 2020a). Furthermore, three positive features including being satisfied with life, optimistic explanatory style, and grit are believed to affect teacher success, considering students’ academic success (Duckworth et al., 2009).

Teacher aggression is said to play a vital role in students’ behavior. Due to teachers’ aggression, students feel distracted from what they do (Montuoro and Lewis, 2018) and feel embarrassed and shameful (Thomas and Montomery, 1998), their self-perceptions can be damaged (Henricsson and Rydell, 2004), and their classmates may start resenting each other (McAuliffe et al., 2009). Post-traumatic stress disorder (Hyman and Snook, 1999) and academic difficulties (Brendgen et al., 2006) have also been associated with teacher aggression. Likewise, students’ sense of responsibility has been reported to reduce (Roache and Lewis, 2011) owing highly to teacher aggression, resulting in misbehavior (Mitchell and Bradshaw, 2013). Moreover, owing to teacher aggression, students’ perception of teacher caring has been believed to diminish (Teven, 2013), leading to lowering teacher effectiveness (Mainhard et al., 2011).

Burnout is a psychological syndrome that causes a person to be under work-related stress for a long time (Maslach, 2003; Wang and Guan, 2020; Derakhshan et al., 2022). It was Freudenberger (1974) who developed this term in psychology for the first time. It was described as a state of exhaustion that occurs due to working too much without giving much attention to one’s own needs (Byrne, 1999). Later, burnout was defined as “a syndrome of emotional exhaustion, depersonalization, and reduced personal accomplishment that can occur among individuals who do people work of some kind” (Maslach and Jackson, 1986, p. 1). Further, Pines and Aronson (1988) made burnout relevant to emotional, mental, and physical exhaustion that can be created due highly to the fact that one has been exposed to emotionally demanding situations for a long time. When one must cope with such prolonged stress which is relevant to work and he fails to be successful in doing it, it can be called burnout (Jennett et al., 2003). Considering teacher burnout, emotional exhaustion is when teachers’ energy is really sapped due to emotional exhaustion. Likewise, another subscale of depersonalization refers to when teachers feel heartless and they are uninterested in their students and their jobs. The third subscale of burnout, low personal achievement is when teachers cannot feel effective or competent to help their students in their learning process (Maslach et al., 2001).

Studies revealed that both individual and environmental factors can cause burnout. There was a thorough study conducted by Skaalvik and Skaalvik (2010) which probed the relationship among burnout, self-efficacy, job satisfaction, and contextual factors. It was found that time pressure and relations with parents are the first and most important predictors of emotional exhaustion and depersonalization. Furthermore, a weak, yet significant association between problems relevant to the discipline regarding the students’ behavior and emotional depletion and depersonalization was found. In another research carried out by Vaezi and Fallah (2011), a negative correlation between emotional intelligence and burnout was reported, meaning that the more emotionally intelligent a person is, the less burnout he experiences. Skaalvik and Skaalvik (2009) also indicated that there is a negative association between all the dimensions of burnout and teacher independence and support. In other words, autonomous, supportive teachers are unlikely to feel burnout. In another research, it was proposed that institutional supervision could predict teacher burnout, which means that they are highly likely to feel burnout (Ghanizadeh and Ghonsooly, 2014) when teachers are supervised by the institute authorities. Moreover, it was revealed by them that self-regulation is negatively and significantly correlated with teacher burnout. Lauerman and König (2016) also disclosed that self-efficacy and pedagogical knowledge are negatively associated with burnout, meaning that the more well-educated and self-efficacious a teacher is, considering the pedagogical knowledge, the less burnout experience they will have.

Grayson and Alvarez (2008) evaluated the impact of school atmosphere (including the relationship between parents and school, administrations, and student behavioral values) on the dimensions of burnout. Some demographic factors including years of experience, gender, age, job satisfaction, and the relationship between students and teachers were also regarded. The findings of the study showed that different factors relevant to school ambiance were correlated with each of the three burnout dimensions. In another study conducted by Eghteasadi Rudi (2011), it was also indicated that low levels of students’ proficiency, lack of advocacy from administration, and second language itself were the crucial reasons for teacher burnout. Moreover, autonomous, self-efficacious, and extroverted teachers resisted burnout in comparison to their counterparts with lower levels of the personal traits mentioned. Furthermore, job satisfaction was found to play a paramount role in all three burnout subscales (Etminan, 2014). In other words, emotional exhaustion and depersonalization were negatively correlated with job satisfaction, while personal accomplishments were positively associated with job satisfaction. Nayernia (2021) believed language proficiency is negatively in line with depersonalization and emotional exhaustion as two subscales of burnout, and it is positively correlated with the personal achievement dimension of burnout. Based on Faskhodi and Siyyari (2018), a significant and negative relationship was found between work engagement and burnout. Furthermore, burnout level was reported to lessen as the years of experience increase. In contrast, teachers’ experience is positively correlated with work engagement.

As a result, these teachers who do not have the capability to channel their negative feelings, such as aggression and burnout, are reported to be less resilient when facing difficulties in the classroom. All in all, teachers with a great positive psychological capital are perceived to manage their negative feelings in a way that it cannot affect the amount of teachers’ success toward their jobs and they would be still motivated enough to challenge their teaching methods every now and then (Derakhshan et al., 2022; Wang et al., 2022c). It has been believed that factors leading to students’ demotivation are self-related, teacher-related, and instruction-related, the lack of positive psychological capital. A study conducted in Chinese universities showed that freshmen and sophomores are more likely to feel demotivated, out of whom freshmen ascribed their demotivation to their teachers because of their teachers’ tedious teaching mood. As opposed to freshmen, sophomores put the blame on the teaching materials, textbooks contents, for example (Wang and Guan, 2020). Accordingly, it should be taken into consideration that there is a link between students’ demotivation and English as a foreign language (EFL) teachers’ negative feelings. In a conceptual study conducted by Wang et al. (2022b), the emphasis that was placed on love or a loving learning atmosphere has been ignored. Therefore, it can be said that both aggression and burnout do not allow a teacher to provide students with love which seems necessary in a fair educational system.

There are some advantages and disadvantages considering the previous studies. Regarding the drawbacks, few studies, if any, to the best of my knowledge thus far, have been done to find the interplay between these three variables of this study. Thus, teachers’ aggression and burnout which are perceived as one of the contributory factors affecting the educational system were to be dealt with positive psychological capital. It is important since the more burned out teachers are, the more aggressive they are. That is, they are less likely to encourage the students to follow their academic goals, and in this way, the learning process cannot be facilitated and, as a result of which, the educational system will not be boosted. Secondly, when teachers feel anxious and burned out, they are less likely to be actively engaged in what they do, and accordingly, it causes them to be less satisfied with their job and feel agitated since they cannot be creative to come up with new ideas for their teaching methods and encouraging their students. Little by little, they may lose inspiration and they fail to be professionally successful. Therefore, it can negatively influence the educational system in which teachers play a pivotal role. That is the reason why the effect of EFL teachers’ aggression and burnout on their professional success has been studied in the current research.

As the review of previous studies revealed, little, to my best knowledge, has been written in EFL context to probe into the association between teacher aggression, burnout, and professional success. Moreover, cultural and geographical factors play a significant role in the results of this study since aggression is really associated with cultural factors (Hofstede, 1986; Wang et al., 2022a). Therefore, this study can be conducted in other countries rather than China in which the current research was carried out. To address this issue, this research strives to discover the associations between teachers’ aggression, burnout, and professional success. In this regard, the following research questions were posed:

1. Are there any significant relationships between Chinese EFL teachers’ aggression, burnout, and their professional success?
2. Do Chinese EFL teachers’ aggression and burnout significantly predict their professional success?

## Materials and methods

### Participants

Of the 430 Chinese EFL teachers whom we approached, a total of 362 teachers (male = 59, 16.3%; female = 303, 83.7%), aged between 25 and 59 (average = 40), volunteered to participate in the present study. All the participants were university EFL teachers, whose teaching experience ranged from 1 to 26 years. Their majors mainly included English literature studies and applied linguistics, with Ph.D. holders (*N* = 11, 3.04%), master’s degree in literature (*N* = 174, 48.07%), and bachelor’s degree in English language teaching (*N* = 121, 33.43%), and others (*N* = 56, 15.47%).

### Instruments

#### Teacher aggression scale

The teacher aggression scale was developed. This instrument included 12 items developed to evaluate teachers’ tendencies to react to learners’ misbehavior aggressively. Those participants replied to each item utilizing a six-point Likert scale which ranges from 1 (not at all descriptive of me) to 6 (very descriptive of me). Reactive, instrumental, and passive aggressions were measured through this scale. To ensure the reliability of the questionnaire administered in this study, the Cronbach’s alpha test was run. It was indicated that the teacher aggression scale (0.88) had satisfactory reliability indices.

#### Teacher burnout scale

To evaluate teachers’ burnout, the teacher version of the Maslach burnout inventory (MBI-ES), designed and validated by Maslach et al. (1996), was exploited. It comprises 22 items evaluating three subscales of burnout, the reduced personal achievement, depersonalization, and emotional exhaustion, a seven-point Likert scale ranging from 0 (never) to 6 (every day). To make sure of the reliability of the questionnaire administered in this study, the Cronbach’s alpha test was run. It was indicated that the teacher burnout scale (0.79) had satisfactory reliability indices.

#### Teacher professional success questionnaire

For the EFL teacher’s professional success to be measured**, the** Characteristics of Successful Language Teachers Questionnaire (CSLTQ) developed and validated by Sadeghi and Babai (2009) was utilized to evaluate teachers’ perceptions of the features of a successful language teacher. The CSLTQ consists of eight factors in the form of 46, five-point Likert-type items ranging from 1 (strongly disagree) to 5 (strongly agree). To guarantee the reliability of the questionnaire administered in this study, the Cronbach’s alpha test was run. It was indicated that the teacher success questionnaire (0.97) had satisfactory reliability indices.

#### Data collection

Based on the EFL teachers’ language competence, the questionnaires we adopted were distributed in the English original through the researcher’s WeChat groups (note: WeChat is a communication app that is popular with all the Chinese people) to get trustworthy data. At the very beginning of the questionnaire, all the participants were provided with their consent to allow their data to be used as research data if their identity information was kept confidential. The present study was carried out in three provinces (Henan, Shanxi, and Zhejiang) in China, beginning in December 2021 and ending in February 2022. Then, we collected more than 400 questionnaires, but only 362 participants’ data were valid to be further analyzed after we gleaned the original data.

## Results

To decide upon the parametric or non-parametric analysis, a test of normality was run. The results are shown in the following:

Table 1 shows the indices of Kolmogorov–Smirnov and shows that the distribution of data was not normal for any of the variables since the p-value is lower than the significance level (*p* = 0.000). Consequently, the non-parametric analysis, namely, Spearman’s rho test, was used.

**TABLE 1:** Test of normality.

	Kolmogorov–Smirnov			Shapiro–Wilk		
	Statistic	Df	Sig.	Statistic	df	Sig.
TA	0.100	362	0.000	0.908	362	0.000
TB	0.101	362	0.000	0.925	362	0.000
TS	0.073	362	0.000	0.948	362	0.000

a. Lilliefors Significance Correction.

### The first research question

The first research question deals with the relationship among three variables of this study (i.e., teacher aggression, teacher burnout, and teacher success) which was calculated through running a Spearman’s rho correlation test.

Table 2 shows the relationship among the Chinese EFL teachers’ aggression, burnout, and success. As seen in this table, there are negative relationships, first, between TA and TS (−0.231) and, second, between TB and TS (−0.122). Moreover, the relationship is significant for both teachers’ aggression (Sig = 0.000) and burnout (Sig = 0.000). It can be concluded that if teachers’ indices of aggression and burnout increase, the index of teachers’ success decreases.

**TABLE 2:** Correlations among Chinese EFL teachers’ aggression, burnout, and success.

			TA^1^	TB^2^	TS^3^
Spearman’s rho	TA	Correlation coefficient	1.000	0.327	−0.231
		Sig. (2-tailed)	.	0.000	0.000
		N	362	362	362
	TB	Correlation coefficient	0.327	1.000	−0.122
		Sig. (2-tailed)	0.000	.	0.000
		N	362	362	362
	TS	Correlation coefficient	−0.231	−0.122	1.000
		Sig. (2-tailed)	0.000	0.000	.
		N	362	362	362

**. Correlation is significant at the 0.01 level (2-tailed).

^1^: Teacher aggression.

^2^: Teacher burnout.

^3^: Teacher success.

### The second research question

The second research question deals with measuring the predictability power of teacher aggression and burnout for teacher success. To this end, a linear multiple regression analysis was performed in the following tables:

The model summary (in Table 3) shows how much of the variance in the dependent variable scores can be explained by the model. Expressed as a percentage, it implies that the model explained 34 percent of the variance in scores from teachers’ success.

**TABLE 3:** Model summary for Chinese EFL teachers’ aggression, burnout, and success.

Model	R	R-square	Adjusted R-square	Std. error of the estimate
1	0.18	0.34	0.029	22.23

a. Predictors: TA, TB.

To evaluate the statistical importance of the findings, it was significant to consider Table 4 labeled ANOVA. This scrutinizes the hypothesis that multiple R in the population equals zero (0). The model achieved statistical significance (Sig = 0.000, meaning that *p* < 0.05).

**TABLE 4:** ANOVA for Chinese EFL teachers’ aggression, burnout, and success.

Model		Sum of squares	Df	Mean square	F	Sig.
1	Regression	6336.12	2	3168.06	6.40	0.000
	Residual	177448.91	359	494.28		
	Total	183785.03	361			

a. Dependent variable: TS.

b. Predictors: TA, TB.

In this investigation, the researcher was fond of making a comparison among the contribution of each independent variable. Hence, the beta values (in Table 5) were utilized. Paying attention to the beta column, it was discovered that the largest beta coefficient was.19 (sig = 0.000), that was ascribed to teachers’ aggression, meaning that this variable was greatly correlated with the dependent variable and it can vividly clarify it when the variance clarified by all other variables in the model was controlled. The beta value for the other variable (i.e., teacher burnout) was also significant (sig = 0.000). It implies that teachers’ burnout, similar to teachers’ aggression, is a significant predictor of teachers’ success.

**TABLE 5:** Coefficients for Chinese EFL teachers’ aggression, burnout, and success.

Model		Unstandardized coefficients		Standardized coefficients	T	Sig.
		B	Std. error	Beta		
1	(Constant)	201.16	7.09		28.33	0.000
	TA	−0.50	0.14	−0.19	−3.53	0.000
	TB	−0.31	0.09	−0.13	−1.67	0.000

a. Dependent variable: TS.

## Discussion

This study scrutinized the relationship between three important variables, Chinese EFL teachers’ aggression, burnout, and their professional success. This is of utmost importance because hardly ever has such a study been conducted before, especially in the context of China. If teachers’ well-being is threatened and their values are violated, they no longer can pursue their jobs. That is the reason why conducting such studies would be of great benefit for both teachers and educational administrators. According to the current investigation, both teachers’ aggression and burnout are negatively and significantly correlated with teachers’ professional success. Teachers with higher levels of aggression and burnout, therefore, have been thought of as less professionally successful because they feel aggressive and burnout, all energy would be sapped, and they are not inclined to support and help their students and allow them to experience a friendly atmosphere in class in which the process of learning is facilitated. The teacher–student relationship is reciprocal; thus, when one feels aggressive, it adversely affects the other and an interactive class would turn into a place with less motivation. Given that, aggression can be encouraged by many factors, including personal problems, not feeling competent enough in the teaching context, and not being up-to-date with the latest facilities or teaching methods that can be utilized, to name a few. Whatever the root cause of the problem is, the ramification of teachers’ feeling aggressive is that they may feel burnout over time, and it directly influences their success.

The results of this study are somewhat in line with some reviewed articles which were mentioned previously in the introduction part. The consequences in the present study showed that there is a negative correlation between both teachers’ aggression and burnout and their professional success. These results support those of other conducted studies (Duckworth et al., 2009; Etminan, 2014; Lauerman and König, 2016; Faskhodi and Siyyari, 2018; Nayernia, 2021). It was reported in Etminan’s (2014) study that varied dimensions of burnout and job satisfaction are negatively associated with each other. It is in congruence with the results found in this study because both teachers’ burnout and teachers’ aggression are regarded as negative feelings, so they are both adversely correlated with teachers’ professional success and their job satisfaction, respectively. According to Lauerman and König (2016), self-efficacy and educational knowledge are negatively aligned with burnout. In this regard, it can be inferred that positive attributes such as self-efficacy in the study mentioned above and professional success which has been proposed in this study can be in a negative relationship with burnout. Likewise, aggression and burnout are adversely consistent with positive features such as professional success in this research. Based on Duckworth et al. (2009), three positive features including being satisfied with life, optimistic explanatory style, and grit are thought to affect teacher success, considering students’ academic success. Therefore, the mentioned study would be in line with the current study in that if the health span of teachers is lowered by some negative factors including aggression and burnout, other aspects of a teacher’s life can be exacerbated, namely, their personal and professional success.

In accordance with what has been proposed by Nayernia (2021) language proficiency is negatively in line with depersonalization and emotional exhaustion as two subscales of burnout, and it is positively correlated with the personal achievement dimension of burnout, another component of burnout. Likewise, it can be proposed that based on the findings of this study, professional success is negatively associated with all dimensions of burnout mentioned above. Based on Faskhodi and Siyyari (2018), a significant and negative relationship was found between work engagement and burnout. Furthermore, burnout level was reported to lessen as the years of experiences increased. In contrast, teachers’ experience is positively correlated with work engagement. It is, therefore, clear that professional success may increase when a teacher is highly experienced and their feeling of burnout reduces which has been proved in the present study.

As mentioned by Wang et al. (2022a), it was indicated that Asian EFL teachers’ psychological well-being and work engagement had a positive impact on their immunity. Moreover, psychological well-being better predicted the teacher immunity than work engagement in Asia. That study discussed in the key role of positive psychology (PP) and emphasized the language teachers’ need for working in a psychologically healthy atmosphere to stay committed to their job and immune to its difficulties. The mentioned study is aligned with the current one because when teachers have a psychologically healthy environment in which they can work, they can be immune to troubles and it may decrease their risk of aggression and burnout in order to feel professionally successful. It was also mentioned by Gregersen et al. (2020) that the most important stressors for teachers by far were time pressure and the juggling of roles, and the greatest uplifts were felt while socialization followed by the stress-reducing activity of “resting”. It is in line with the current study because aggression may increase when teachers are pressed for time, and in the long run, feeling burned out may raise as well. Socializing may decrease the risk of feeling burned out and heighten teachers’ success.

## Conclusion, pedagogical implications, and limitations

The conclusion that can be drawn from this study is that the role of Chinese EFL teacher aggression in their burnout and professional success has been found to be significantly important. It should be highlighted that teacher aggression and burnout are both good predictors of teachers’ professional success. The results of this study can be of high interest for teachers themselves and educational authorities. It can be taken into consideration for the administrations that many contributory factors, such as “job satisfaction, supporting teachers and allowing them to feel autonomous in the way they teach and manage the class, providing them with the cutting-edge facilities through which both teachers’ motivation and creativity, can be enhanced, and providing them with in-service training from time to time” can cause teachers to feel less aggressive and burned out even if the underlying cause of these feelings may be rooted in some personal problems. On the other hand, owing to the stressful, demanding job that a teacher has, they may experience such prolonged feelings, and they had better be aware of the point that if care is given, they will not last for a long time which is worrisome for many teachers. Furthermore, they could be cognizant of different symptoms of these feelings, and when they know the exact underlying cause of the problem, solutions wisely can be put forward to address the problem. It is highly crucial for a teacher to be professionally successful and acclaimed by his students because in this respect they would be more motivated to increase their pedagogical knowledge and modify their problematic behavior that may mostly affect students’ performance and their academic success.

From another point of view, it is extremely important to shift attention to the point that these three variables help the educational system to grow. When it comes to the educational system, it can be divided into three categories: students, teachers, and teacher educators. Without a shadow of a doubt, all the factors including the educational ambiance and the facilities can fall under these categories as well. It is vividly clear that teachers’ professional success plays a paramount role in enhancing students’ knowledge; when teachers are optimistic and hopeful both about what has been done and taught by them and about their students’ achievements, students can find the motivation to learn more and more in a friendly atmosphere where the learning process is facilitated, for example. Similarly, if teachers are satisfied with greater success, they are more likely to feel committed to what they teach and the materials and teaching methods they use to convey their message while teaching. Since problems are perceived as something soluble by them, not the barriers by which they can be stopped from what they are focusing on. So it definitely ameliorates the educational system. From another point of view, teachers’ professional success can highly improve the educational system because the more engaged a teacher is in their work, the better atmosphere can be built both for teaching and learning process where both teachers and students can get benefit. Teachers, who are less aggressive and feel less burned out, are professionally successful, and they can urge students more to reach their apex of learning and achieve their goals which have been planned. Considering the opposite, students are not motivated enough to find the learning process productive and follow their goals. They may feel demotivated and find no meaning in this process which otherwise could be vibrant. Thus, this study can enhance the educational infrastructures.

One of the practical implications of this study would be the fact that how aggression can be reduced and how burnout would be prevented for teachers to be professionally successful. Aggression would be lessened if teachers’ confidence and buoyancy are raised. Likewise, feeling burned out can also be prevented when they are motivated enough and enthusiastic to be actively engaged in the teaching process which causes students to feel involved as well.

Finally, this study is limited in some ways. First, China was the only context where this research was conducted; consequently, the same study can be carried out by avid teachers in any other country since different cultures can tremendously affect the consequences reported. Secondly, the reasons behind aggression were not dealt with in the current study; therefore, they can be probed with their relations to professional success in the future studies. Thirdly, even though quantitative studies are believed to be more reliable in that they are objective, qualitative investigations can be conducted to find some deeper results since teachers can report everything precisely by keeping a diary and journal over time, and with the passage of time, more precise details can be revealed about teachers’ feelings. All in all, every study has its own limitations even though it paves the way for further studies.

## Data availability statement

The original contributions presented in this study are included in the article/supplementary material, further inquiries can be directed to the corresponding author.

## Ethics statement

The studies involving human participants were reviewed and approved by Yancheng Teachers University Academic Ethics Committee. The patients/participants provided their written informed consent to participate in this study.

## Author contributions

The author confirms being the sole contributor of this work and has approved it for publication.
